# Recent advances in understanding and treating acute respiratory distress syndrome

**DOI:** 10.12688/f1000research.15493.1

**Published:** 2018-08-20

**Authors:** Rahul S. Nanchal, Jonathon D. Truwit

**Affiliations:** 1 Pulmonary and Critical Care Medicine, Medical College of Wisconsin, Milwaukee, WI, USA; 2 Pulmonary and Critical Care Medicine, Froedtert & Medical College of Wisconsin, Milwaukee, WI, USA

**Keywords:** ARDS, acute respiratory distress syndrome

## Abstract

Acute respiratory distress syndrome (ARDS) is a clinically and biologically heterogeneous disorder associated with many disease processes that injure the lung, culminating in increased non-hydrostatic extravascular lung water, reduced compliance, and severe hypoxemia. Despite enhanced understanding of molecular mechanisms, advances in ventilatory strategies, and general care of the critically ill patient, mortality remains unacceptably high. The Berlin definition of ARDS has now replaced the American-European Consensus Conference definition. The recently concluded Large Observational Study to Understand the Global Impact of Severe Acute Respiratory Failure (LUNG-SAFE) provided worldwide epidemiological data of ARDS including prevalence, geographic variability, mortality, and patterns of mechanical ventilation use. Failure of clinical therapeutic trials prompted the investigation and subsequent discovery of two distinct phenotypes of ARDS (hyper-inflammatory and hypo-inflammatory) that have different biomarker profiles and clinical courses and respond differently to the random application of positive end expiratory pressure (PEEP) and fluid management strategies. Low tidal volume ventilation remains the predominant mainstay of the ventilatory strategy in ARDS. High-frequency oscillatory ventilation, application of recruitment maneuvers, higher PEEP, extracorporeal membrane oxygenation, and alternate modes of mechanical ventilation have failed to show benefit. Similarly, most pharmacological therapies including keratinocyte growth factor, beta-2 agonists, and aspirin did not improve outcomes. Prone positioning and early neuromuscular blockade have demonstrated mortality benefit, and clinical guidelines now recommend their use. Current ongoing trials include the use of mesenchymal stem cells, vitamin C, re-evaluation of neuromuscular blockade, and extracorporeal carbon dioxide removal. In this article, we describe advances in the diagnosis, epidemiology, and treatment of ARDS over the past decade.

## Introduction

Described over 50 years ago ^1^, acute respiratory distress syndrome (ARDS) remains a devastating manifestation of heterogeneous disease processes that injure the lung and cause non-hydrostatic pulmonary edema ^2, 3^. The clinicopathological correlates include severe inflammatory injury to the alveolar–capillary barrier ^4^, surfactant depletion ^5^, and loss of aerated lung tissue, resulting in refractory hypoxemia, reduced lung compliance ^6^, and increases in venous admixture ^7^ and dead space ^8^. Despite considerable progress in the understanding of molecular mechanisms underpinning the biology of ARDS, the armamentarium of therapies remains limited and mortality rates are approximately 40% ^9^. In this review, we describe advances in the diagnosis, epidemiology, and treatment of ARDS over the past decade. Where applicable, we provide historical background and highlight areas of uncertainty and directions for future research.

## Diagnosis

The first concerted effort to define ARDS as a clinical syndrome appeared in 1988, when Murray *et al*. assigned points to the chest radiograph, partial pressure of oxygen/fraction of inspired oxygen (P/F) ratio, applied positive end expiratory pressure (PEEP), and lung compliance to create a lung injury score that ranged from 0 to 4 ^10^. Until recently, the most widely accepted definition was that from the American European Consensus Conference (AECC) in 1994 ^11^. The AECC defined ARDS as the acute onset of hypoxemia with bilateral infiltrates on a frontal chest radiograph, with no clinical evidence of left atrial hypertension (or pulmonary artery wedge pressure of greater than or equal to 18 mmHg when measured) and a P/F ratio of less than or equal to 200 mmHg. Acute lung injury (ALI) was also defined using the same criteria but a P/F threshold of 300 mmHg; thus, ARDS was a subset of patients with ALI. Although this consensus definition better enabled comparative studies of epidemiology and mortality and enrollment in clinical trials, it was hampered by many limitations. Key amongst the limitations were the uncertainty of timing of the insult leading to ARDS, confusion surrounding the ALI category, ambiguity surrounding the use of P/F ratio relative to the application of PEEP which may modify this ratio, marked inter-observer variation in the interpretation of chest radiography, and controversies in excluding volume overload or heart failure as the primary cause of respiratory failure. The current Berlin definition of ARDS addresses these limitations ^12^. It specifies an acute time frame for the development of ARDS (within one week of known clinical insult or new or worsening respiratory symptoms), stipulates minimum ventilator settings (PEEP of 5 cm H _2_O or more), and clarifies chest radiography criteria as well as the adjudication of respiratory failure from volume overload or heart failure. Furthermore, the Berlin definition removes the ALI term and classifies ARDS into three categories (mild, moderate, and severe) to facilitate prognostication. However, the prognostic ability is limited, partly owing to the heterogeneity of disease processes that culminate in ARDS and an individual clinician’s inaccurate discriminatory ability of disease trajectory ^12^. Reclassifying ARDS at day two (24 hours after first recognition) added little to the predictive value for mortality, lending credence to the fact that most patients die from complications of critical illness rather than ARDS itself ^13^. Finally, as resource-poor environments have limited accessibility to mechanical ventilators, arterial blood gases, and chest radiographs, the prevalence and severity of ARDS may be underestimated. To overcome these difficulties, the Kigali modification of the Berlin definition of ARDS has been proposed ^14^. It substitutes S/F ratio of less than or equal to 315 for the P/F ratio (where S is the peripheral capillary oxygen saturation as measured by a pulse oximeter), eliminates the PEEP criteria, and accommodates the evaluation of bilateral lung opacities via lung ultrasound ^14^. A recent attempt at external validation of the Kigali criteria resulted in good sensitivity but moderate specificity predominantly because of false positive lung ultrasounds ^15^.

### Diagnostic uncertainty and future research

Although the Berlin definition provides guidance for the interpretation of chest radiographs and determination of the origin of pulmonary edema, these two areas are still dependent on clinician interpretation and may lead to under/over recognition and under/over treatment of ARDS. The recently developed Radiographic Assessment of Lung Edema (RALE) score may be a novel tool to predict severity, outcomes, and response to therapy in ARDS. In the derivation cohort of this study, the RALE score was correlated with lung weight from excised lungs in deceased organ donors ^3^. Prior to widespread use of this score, extensive validation is required. Use of lung ultrasound for the diagnosis of ARDS may be a promising tool but needs further study. Sensitive and specific biomarkers of ARDS, if available, may aid in earlier and more accurate diagnosis.

## Epidemiology

Many studies have attempted to discern the incidence, outcomes, and population metrics of ARDS ^14, 16– 19^. Despite standardized definitions, most are hampered by inter-observer variability in ascertainment of cases, variations in validity of case definitions, geographic differences in availability of medical resources such as intensive care unit (ICU) beds, heterogeneity of risk factors amongst populations, and problems with determining mortality that can be directly attributed to ARDS, leading to inconsistencies of epidemiological measures ^20– 22^. The Large Observational Study to Understand the Global Impact of Severe Acute Respiratory Failure (LUNG-SAFE) is the latest cross-sectional study that provides data on the epidemiology of ARDS ^9^. Of the 29,144 patients enrolled in ICUs across 50 countries, the period prevalence of ARDS was 10.4%. Of the 12,906 patients who received mechanical ventilation, 23.4% fulfilled the Berlin definition of ARDS. There was over a twofold geographic variation in the ICU incidence of ARDS over a four-week period despite seasonal adjustment and standardization of case definitions. Unadjusted ICU and hospital mortality from ARDS was 35.3% and 40%, respectively.

With attempts to understand why virtually every clinical therapeutic trial in ARDS has failed, attention has shifted to comprehend the clinical and biological heterogeneity of ARDS as a syndrome. Such attempts have been made in other complex syndromes such as asthma wherein identification of endotypes (subtype of a condition, which is defined by a distinct functional or pathophysiological mechanism) has led to testing and subsequent success of novel therapies ^23, 24^. Using a sophisticated mathematical approach called latent class analysis, investigators were able to identify two clinically and biologically distinct subphenotypes of ARDS in independent analyses of three large ARDS cohorts, all from the National Heart, Lung, and Blood Institute’s (NHLBI) ARDS Network: the ARMA trial of low tidal volume ventilation, the ALVEOLI trial of low versus high PEEP, and the Fluid and Catheter Treatment Trial of liberal versus conservative fluid management in the acute lung injury/ARDS ^25^. These two subphenotypes have distinctly different biomarker profiles and clinical courses and respond differently to the random application of PEEP ^25^ and fluid management strategies ^26^. The “hyper-inflammatory” phenotype as opposed to the “hypo-inflammatory” phenotype is characterized by higher concentrations of plasma inflammatory biomarkers, higher prevalence of vasopressor use, lower serum bicarbonate levels, and higher prevalence of sepsis ^25^. Outcomes of mortality, ventilator-free days, and organ failure-free days were all worse in the “hyper-inflammatory” phenotype. Furthermore, response to treatment was vastly different in the two subphenotypes: high PEEP improved outcomes only in the hyper-inflammatory phenotype, while liberal fluid management worsened mortality ^25, 26^. Conversely, a conservative fluid management strategy was harmful in the hypo-inflammatory phenotype. This differential response to therapy suggests that these subphenotypes may actually be endotypes of ARDS. Data suggest that these two phenotypes can be identified with remarkable accuracy using just four biomarkers: interleukin-6, interferon gamma, angiopoietin 1/2, and plasminogen activator inhibitor-1 (area under the receiver operator curve = 0.98) ^27^. It has now been demonstrated that patients assigned to a particular subphenotype on day 0 stay within the same subphenotype at day 3 ^28^. This finding provides further evidence of fundamental biological differences between phenotypes that are not governed by time of measurement and supports the notion that these subphenotypes can realistically be targeted for enrollment in clinical trials.

### Uncertainties in epidemiology and future research

The epidemiology of ARDS suffers from the lack of a true diagnostic test. The discovery of phenotypes/endotypes based on physiology and biology will likely provide mechanistic insight rather than definitions of a heterogeneous syndrome. Future investigations should refine clinical, radiographic, physiological, molecular, and genetic differences in phenotypes to better understand the pathobiology of ARDS and develop novel therapies.

## Therapy

Despite decades of investigation, therapies for ARDS remain remarkably limited. Mechanical ventilation is the mainstay of treatment with physiological strategies targeted to reduce ventilator-induced lung injury (VILI) by minimizing lung stress and strain ^29^. In this section, we first describe advances in strategies that minimize VILI and then discuss adjunctive and pharmacological therapies.

### Ventilator-induced lung injury

An era of research into how mechanical ventilation could injure the lungs of ARDS patients *de novo* culminated in the NHLBI ARDS Network ARMA trial that made low tidal volume ventilation the cornerstone strategy of provision of this therapy worldwide ^30^. Clinically, VILI has two major manifestations, volutrauma and atelectrauma, both intricately linked to lung stress and strain ^29, 31^. Lung stress refers to transpulmonary pressure (alveolar pressure – pleural pressure), and strain refers to the change in lung volume indexed to functional residual capacity of the ARDS lung at zero PEEP. Thus, lung stress is nothing but the specific lung elastance multiplied by lung strain ^29, 31^. Volutrauma therefore refers to excessive generalized stress/strain on the lung ^29, 31^. Sophisticated CT scan studies and elegant physiological investigations have shown that lung injury in ARDS is heterogeneous, with injured or atelectatic areas adjacent to relatively normal aerated areas ^32^. These interfaces between collapsed and open areas may affect up to 40% of the lung and act as local “stress raisers”, i.e. local stress and strain at such interfaces are greatly multiplied and may far exceed average global stress and strain ^32, 33^. A recent study by Gattinoni *et al*. demonstrated that focal stresses may be multiplied by a factor of two ^32^. Minimizing VILI thus targets reducing volutrauma (reduction of global stress and strain) and reducing atelectrauma through the “open lung approach” (open the lung and keep it open through the respiratory cycle). A newer concept to reduce VILI is “permissive atelectasis”, i.e. the application of lower PEEP levels combined with low tidal volumes to prevent repeated opening and closing of alveoli ^34^. Lowering airway pressures also has the dual benefit of minimizing overdistension of the aerated areas and mitigating negative hemodynamic consequences. This strategy has some experimental support: reduced injury and inflammation in the atelectatic areas ^35, 36^. However, it needs to be tested in large clinical trials before it can be recommended.

### Trials targeting the open lung approach

***High-frequency oscillatory ventilation trials.*** The theoretical concept underpinning high-frequency oscillatory ventilation (HFOV) is the use of very small tidal volumes oscillating around a very high mean airway pressure, thus limiting both volutrauma and atelectrauma. Two large randomized controlled trials (RCTs) tested this concept. The Oscillation in ARDS (OSCAR) trial compared HFOV to usual ventilator strategy but could not demonstrate any differences in 30-day mortality ^37^. The Oscillation for Acute Respiratory Distress Syndrome Treated Early (OSCILLATE) trial was stopped prematurely secondary to safety concerns ^38^. OSCILLATE compared HFOV to conventional ventilation with relatively higher PEEP levels and demonstrated a much higher mortality associated with HFOV likely secondary to adverse hemodynamic consequences of high mean airway pressures demonstrated by the higher use of vasoactive agents in the HFOV group. These results do not support the routine use of HFOV in ARDS; however, a patient-level meta-analysis does indicate that HFOV may be beneficial in patients with very severe hypoxemia (P/F ratio of less than 64 mmHg) ^39^.

***Recruitment maneuver and high positive end expiratory pressure trials.*** A lung recruitment maneuver comprises the application of high airway pressures and/or PEEP for a variable duration of time to open collapsed lung units. A recruitment maneuver is usually followed by a trial of PEEP decrements to adjudicate the minimum PEEP required to keep the lung open. Multiple trials have failed to demonstrate benefit of using recruitment maneuvers in ARDS patients ^40, 41^. A recent Cochrane review of trials (n = 10; 1,658 patients) involving recruitment maneuvers in ARDS could not find differences in 28-day or hospital mortality ^42^. Another recent meta-analysis concluded that higher PEEP was unlikely to improve clinical outcomes among unselected patients with ARDS ^43^. The most recent RCT of recruitment maneuvers in ARDS was the multicenter Alveolar Recruitment for ARDS Trial (ART). This study randomized patients with moderate or severe ARDS to one of two strategies: one with a lung recruitment maneuver and PEEP titration determined by the best respiratory system compliance or one with a control approach of low PEEP ^44^. There was a significant increase in 28-day and 6-month mortality in the recruitment maneuver arm. This arm also experienced fewer ventilator-free days and a heightened risk of barotrauma compared to the conventional arm. Aggregated, results from these investigations currently do not support the routine use of recruitment maneuvers in unselected ARDS patients. Results of these clinical trials also support the idea that reducing tidal volume to decrease global stress and strain appears to be more important than preventing atelectrauma and that the application of high airway pressures in the form of recruitment maneuvers and PEEP predisposes the lung to volutrauma.

### Airway pressure release ventilation

The use of this mode of mechanical ventilation has proliferated despite lacking evidence for benefit or head-to-head comparison with conventional low tidal volume ventilation. A recent small single-center trial of 138 patients with ARDS randomized patients to airway pressure release ventilation (APRV) versus conventional ventilation and reported more ventilator-free days in the APRV arm (median 19 versus 2) ^45^. The results of this trial should be interpreted with caution, as most outcomes of patients in the conventional ventilator arm were much worse than those reported in any NHLBI ARDS trials.

### Adjuncts to ventilator strategies to reduce lung stress and strain

***Neuromuscular blockade.*** By facilitating ventilator synchrony, neuromuscular blockade, especially in early ARDS, may reduce VILI by ensuring low delivery of low tidal volume (prevention double triggering), preventing loss of PEEP by active exhalation (prevention of atelectrauma), and controlling the high ventilation demand that is inherent to severely injured lungs and respiratory drive associated with hypercapnia during low tidal volume ventilation as well as through a direct anti-inflammatory effect ^46^. In a multicenter trial involving 340 patients with severe ARDS (P/F ratio of less than 150 with PEEP of 5 cm H _2_O or more), patients randomized to neuromuscular blockade with cisatracurium besylate for 48 hours had a 90-day risk of death that was much less than their non-paralyzed counterparts (hazard ratio 0.68) after adjustment for confounding variables ^47^. Importantly, the rates of ICU acquired weakness did not differ between the two groups. It is therefore reasonable to consider neuromuscular blockade in patients with uncontrolled respiratory drives or experiencing ventilator asynchrony after adequate sedation.

***Prone positioning.*** In ARDS, dependent lung regions become consolidated as the lung collapses on its own weight. This phenomenon is exacerbated by the weight of the heart compressing adjacent lung regions. Prone positioning ameliorates these phenomena, as the weight of the lung is suspended from a larger horizontal dorsal chest wall. This homogenizes ventilation to lung areas, reduces stress and strain, and alleviates stress raisers. Further, perfusion is more evenly distributed, leading to better V/Q matching and consequently better gas exchange ^48, 49^. Despite sound physiological rationale and elegant pre-clinical models, prior to the most recent trial, several studies utilizing prone position in ARDS were negative ^50, 51^. The prone position in severe ARDS (PROSEVA) trial recruited 466 patients with severe ARDS (P/F of 150 mmHg or less) and randomized them to prone position for at least 16 hours a day or conventional therapy ^52^. There was a marked decrement in the risk of death in the prone position arm (hazard ratio = 0.44) with no differences in adverse outcomes except the number of cardiac arrests in the supine position. Based on strong biological plausibility and results of the large clinical trial, it is prudent to utilize prone positioning for patients with severe ARDS and strongly consider it for patients with moderate ARDS. In fact, clinical guidelines for mechanical ventilation endorsed by the American Thoracic Society, European Society of Intensive Care Medicine, and Society of Critical Care Medicine provide a strong recommendation to use prone position for more than 12 hours a day in patients with severe ARDS ^53^.

***Extracorporeal membrane oxygenation.*** Used mainly as a rescue therapy, the use of extracorporeal membrane oxygenation (ECMO) for patients with ARDS remains controversial. Many centers advocate early use and prefer it to prone positioning ^54^. The rationale is the ease of delivery of lung protective ventilation because associated gas exchange abnormalities are corrected via the extracorporeal gas exchanger. Several reports during the H1N1 pandemic in 2009 alluded to benefit ^55, 56^, but none of these were randomized investigations. The recently concluded ECMO to Rescue Lung Injury in Severe ARDS (EOLIA) trial randomly assigned patients with very severe ARDS, (P/F of 50 mmHg or less for more than three hours, a P/F of less than 80 mmHg for more than six hours, or an arterial blood pH of less than 7.25 with a PaCO _2_ of 60 mmHg or more for more than six hours) to receive immediate veno-venous ECMO (ECMO group) or continued conventional treatment (control group) ^57^. Crossover to ECMO was possible for patients in the control group who had refractory hypoxemia. The trial was halted prematurely because it was determined that the futility threshold had been crossed. Although there were no differences in mortality, the results were complicated by a large number of patients (28%) in the control group who crossed over to the ECMO group because of refractory respiratory failure. The results, however, did not show that routine use of ECMO in severe ARDS is superior to reserving ECMO as a rescue maneuver. Therefore, it may be prudent to utilize other evidence-based recommendations such as neuromuscular blockade and prone positioning prior to the initiation of ECMO.

***Non-invasive ventilation.*** A single-center RCT of helmet non-invasive ventilation and face mask non-invasive ventilation in patients with ARDS who were already receiving face mask non-invasive ventilation for at least 8 hours was stopped early for efficacy after 83 of a planned 206 patients were enrolled ^58^. Patients receiving helmet non-invasive ventilation had significantly lower rates of endotracheal intubation, more ventilator-free days, and a remarkably better mortality than patients with face mask ventilation. The results of this trial should be interpreted with caution; the face mask arm had a mortality of 56% at 90 days as compared to 35% in the helmet arm, which is much higher than recent NHLBI ARDS cohorts and other epidemiological ARDS studies. This suggests that large differences in mortality may have been secondary to failure of timely intubation, especially in the face mask arm. The LUNG-SAFE study found that in patients with severe ARDS the application of non-invasive ventilation was associated with worse mortality ^59^.

***Pharmacological therapies.*** The clinical landscape in ARDS therapy has been fraught with failure of therapeutic trials. Most recent amongst such failures are the use of statins, beta-2 adrenergic agents, and keratinocyte growth factor (KGF) ^60– 63^. A multicenter trial of aspirin to prevent ARDS ^64^ in high-risk patients (lung injury prevention score over four) ^65^ also failed to reduce the incidence of ARDS. A promising new paradigm in ARDS is the use of cell-based therapies to accelerate injured lung epithelium and endothelium and enhance the clearance of edema fluid. Two phase I trials of mesenchymal stem/stromal cells (MSCs) in ARDS did not report any safety events ^66, 67^, and a phase 2a trial is currently ongoing.

The NIH NHLBI PETAL (prevention and early treatment of ALI) network is presently conducting two RCTs: vitamin D to improve outcomes by leveraging early treatment (VIOLET) and re-evaluation of systemic early neuromuscular blockade (ROSE). Other noteworthy ongoing trials include vitamin C infusion for treatment in sepsis-induced ALI (CITRIS-ALI), two studies looking at vitamin C in combination with corticosteroids and thiamine, liberal oxygenation versus conservative oxygenation in ARDS (LOCO2), protective ventilation with veno-venous lung assist in respiratory failure (REST), and the Strategy of Ultraprotective Lung Ventilation with Extracorporeal CO _2_ Removal for New-Onset Moderate to Severe ARDS (SUPERNOVA).
